# Influence of inpatient withdrawal treatment on drug safety in alcohol use disorder — a quasi-experimental pre-post study

**DOI:** 10.1186/s12888-024-06188-y

**Published:** 2024-10-25

**Authors:** Sebastian Schröder, Martin Schulze Westhoff, Stefan Bleich, Henry Bode, Konstantin Fritz Jendretzky, Benjamin Krichevsky, Alexander Glahn, Johannes Heck

**Affiliations:** 1 Department of Psychiatry, Social Psychiatry and Psychotherapy, Hannover Medical School, Carl-Neuberg-Str. 1, 30625 Hannover, Germany; 2 Department of Neurology, Hannover Medical School, Hannover, Germany; 3 Institute for General Practice and Palliative Care, Hannover Medical School, Hannover, Germany; 4 Institute for Clinical Pharmacology, Hannover Medical School, Hannover, Germany

**Keywords:** Alcohol use disorder, Drug safety, Alcohol–medication interactions, Potentially inappropriate medications, Drug–drug interactions

## Abstract

**Objective:**

Most patients with alcohol use disorder (AUD) regularly take medication. Alcohol interacts negatively with many commonly prescribed medications. Little is known about whether the risk of potential alcohol-medication and drug-drug interactions increases or decreases in patients with AUD during inpatient withdrawal treatment. The aim of our study was to determine the prevalence and characteristics of potential alcohol-medication and drug-drug interactions in patients with AUD before and after withdrawal treatment in an addiction unit.

**Design:**

Prospective monocentric quasi-experimental pre-post study.

**Methods:**

Medication records before and after withdrawal treatment were analyzed and screened for potential alcohol-medication (pAMI) and drug-drug interactions (pDDI) using the drugs.com classification and the AiD*Klinik*^®^ electronic interaction program, respectively.

**Results:**

We enrolled 153 patients with AUD who were treated in an addiction unit of a university hospital in Germany. Of these, 67.3% experienced at least one pAMI before and 91.5% after withdrawal treatment. In total, there were 278 pAMIs classified as “mild,” “moderate,” or “severe” before and 370 pAMIs after withdrawal treatment. Additionally, there were 76 pDDIs classified as “moderate,” “severe,” or “contraindicated combinations” both before and after withdrawal treatment.

**Conclusion:**

The risk of exposure to pAMIs and pDDIs increases during inpatient withdrawal treatment in patients with AUD. Improvements in the quality of prescribing should particularly focus on the use of antihypertensives and opioids.

## Introduction

Alcohol use disorder (AUD) and associated alcohol consumption represent prominent contributors to global morbidity and mortality [1]. Beyond societal implications such as stigma, individuals suffering from alcohol dependence face a spectrum of somatic risks, including cardiovascular, hepatic, and pancreatic diseases [2–4]. Moreover, psychiatric comorbidities (e.g., depression and personality disorders) are prevalent among patients with AUD [5, 6]. The presence of multiple diagnoses often necessitates pharmacotherapy, resulting in frequent polypharmacy among individuals with AUD [7]. However, polypharmacy poses a significant risk for drug-drug interactions (DDIs) and adverse drug reactions (ADRs), which may entail frequent emergency department admissions and hospitalizations [8–10]. 

In addition, people with AUD have an increased susceptibility to potential alcohol-medication interactions (pAMIs) during alcohol consumption, which pose a significant risk to their health [11, 12]. Such interactions can trigger alterations in alcohol metabolism, potentially leading to several adverse outcomes, including increased sedation, hypoglycemic episodes, orthostatic hypotension, increased susceptibility to gastrointestinal bleeding and hepatotoxicity [11]. Not all alcohol-medication interactions (AMIs) are dose-dependent; however, there is evidence of a positive association between higher levels of alcohol consumption and the likelihood of AMIs [13, 14]. In addition, alcohol-related ADRs are more severe than non-alcohol-related ADRs and often require hospitalization [15]. Furthermore, empirical evidence suggests that even moderate alcohol consumption in combination with concomitant medication use may increase the incidence and severity of ADRs [16]. 

Despite existing research examining the prevalence of pAMIs at a population level, there remains a notable gap in the literature with regard to studies examining the prevalence and characteristics of pAMIs in people diagnosed with AUD [14, 17]. In addition, to the best of our knowledge, there are no studies investigating whether qualified inpatient withdrawal treatment is a risk factor for medication safety in AUD [15, 18]. 

Given the increased susceptibility of patients with AUD to ADRs, special attention must be paid to the appropriate prescribing of medications in their medical care. The aim of this study was therefore to prospectively analyze the influence of changes in prescribed medication during inpatient withdrawal treatment on pAMIs and pDDIs in patients treated with an AUD at a German university hospital over a period of one year.

## Methods

### Study design and eligibility criteria

The study was conducted as a prospective quasi-experimental pre-post study. Patients were included in the study, if (i) they were treated in the addiction unit of the Department of Psychiatry, Social Psychiatry and Psychotherapy of Hannover Medical School between February 2023 and January 2024, (ii) if they suffered from AUD, (iii) if there were changes to their medication (prescribing and/or deprescribing), and (iv) if they or their legal representative had provided written informed consent that patient-related data can be used for this study. Hannover Medical School is a large university hospital and tertiary care referral center in northern Germany. The addiction-specific unit is specialized in the treatment and care of patients with substance use disorders. All patients were inpatients. There were no specific exclusion criteria.

### Identification of demographic data

Demographic characteristics, i.e., age, sex, and psychiatric as well as medical diagnoses, were obtained from patient records. Psychiatric diagnoses were defined using the ICD-10 criteria. For comparability they were subsumed into relevant overarching clinical categories such as “depression” or “dementia” (Supplementary methods).

### Medication evaluation tools

Drug prescriptions were analyzed by an interdisciplinary team of experts in psychiatry and clinical pharmacology. To this end, the drugs.com classification [18] (Drugsite Trust, Auckland, New Zealand) and the electronic drug interaction program AiD*Klinik*^®^ (Arzneimittel-Informations-Dienste, Dosing GmbH, Heidelberg, Germany) were utilized for the evaluation of pAMIs and pDDIs, respectively.

Drugs.com provides information on possible interactions between different drugs and psycho-tropic substances including alcohol. This comprises data on the severity of the interaction, possible ADRs and precautions to take. The database contains information on 525 drugs possibly involved in pAMIs. Thirty-seven of the pAMIs are classified as severe, 477 as moderate, and 11 as minor.

Patients’ medications were screened for pDDIs using the electronic drug interaction program AiD*Klinik*^®^. Only pDDIs classified as “moderate”, “severe”, or “contraindicated combination” by AiD*Klinik*^®^ were included in the statistical analysis.

### Statistics

All statistical analyses were conducted with IBM^®^ SPSS^®^ Statistics 28 (Armonk, New York, USA). Descriptive statistical methods were used to summarize the data. Absolute and relative frequencies were calculated for categorical variables. Quantitative variables were checked for normal distribution with the Shapiro–Wilk test and by inspection of histograms and Q–Q plots. Since all quantitative variables were not normally distributed, medians with interquartile ranges (IQRs) were reported instead of means and standard deviations. For comparison of the number of drugs before and after withdrawal treatment, Wilcoxon signed-rank tests were applied. Additionally, we compared patients affected by at least one pAMI or at least one pDDI (hereon referred to as patients with pAMIs and patients with pDDIs, respectively) with patients not affected by pAMIs or pDDIs (hereon referred to as patients without pAMIs and patients without pDDIs, respectively). The proportions of patients with pAMIs and the proportions of patients with pDDIs before and after inpatient withdrawal treatment were analyzed with the McNemar test. Due to the exploratory nature of our study, no adjustments were made for multiple testing.

## Results

### Study population

**Fig. 1:**
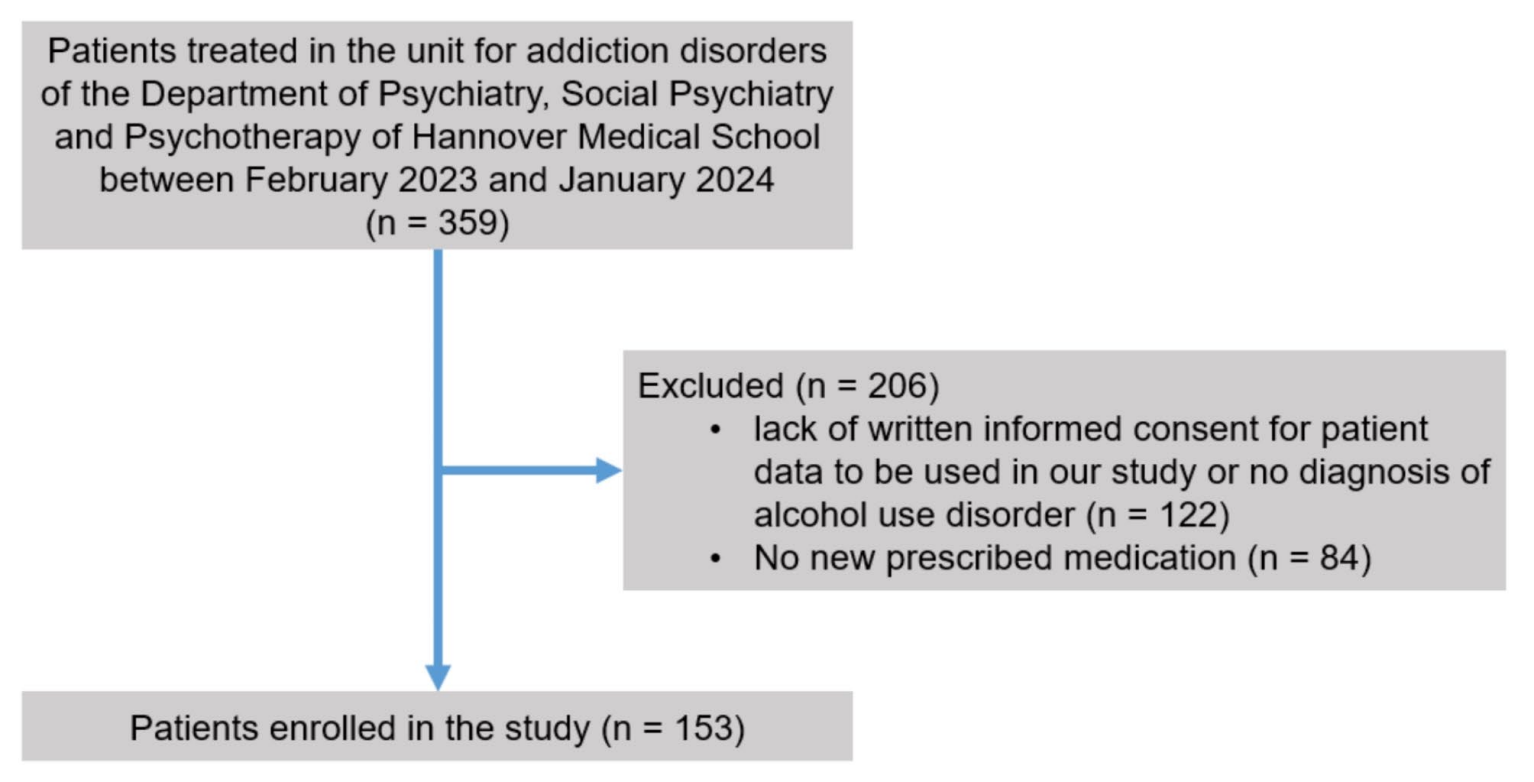
Flow of patient selection

A total of 359 patients were treated in the addiction unit from February 2023 to January 2024, of which 153 fulfilled the inclusion criteria and were enrolled in the study (male 67.3%; female 32.7%) (Fig. 1). The median age of the study population (*n* = 153) was 47 years (IQR 36–54; minimum 19; maximum 80). Nearly half of the study population suffered from comorbid depression (44.4%), and nearly one fifth of the study population had arterial hypertension (18.6%) (Table 1). Other common psychiatric comorbidities included cannabis use disorder (22.2%), personality disorders (21.6%), and cocaine use disorder (15.7%).

**Table 1:** Characteristics of the study population (*n* = 153)

Variables	*n*	%
Sex		
Female	50	32.7
Male	103	67.3
**Psychiatric diagnoses** ^**a**^		
Alcohol use disorder	153	100
Opioid use disorder	17	11.1
Cannabis use disorder	34	22.2
Sedative use disorder	12	7.8
Cocaine use disorder	24	15.7
Multiple substance use disorder	14	9.2
Depression^b^	68	44.4
Bipolar affective disorder^c^	2	1.3
Schizophrenic disorders^d^	5	3.3
Personality disorder^e^	33	21.6
Post-traumatic stress disorder	17	11.1
Dementia^f^	2	1.3
Other psychiatric disorder(s)	72	47.1
**Somatic diagnoses** ^**a**^		
Arterial hypertension	30	19.6
Coronary heart disease	1	0.7
Chronic heart failure	3	2.0
Status post stroke (ischemic or hemorrhagic)	3	2.0
Type-2 diabetes mellitus	14	9.2
Chronic obstructive pulmonary disease	9	5.9
Hypothyroidism	2	1.3
Other somatic disorder(s)	101	66.0

^a^Patients could have more than one diagnosis; ^b^ICD-10 F32, F33; ^c^ICD-10 F31; ^d^ICD-10 F06.2, F20; ^e^ICD-10 F60; ^f^ICD-10 F00, F01, F02, F03

*Abbreviations*ICD-10, International Statistical Classification of Diseases and Related Health Problems 10th Revision PTSD, post-traumatic stress disorder

### Potential alcohol-medication interactions

Patients received significantly more medication after inpatient withdrawal treatment (median of 2 (IQR 0–4) medications before and 3 (IQR 2–5) medications after withdrawal treatment treatment (*p* < 0.001 (Wilcoxon signed-rank test))). Before detoxification 67.3% of all patients were affected by at least one pAMI and 91.5% thereafter (*p* < 0.001 (McNemar test)). Overall, there were 278 pAMIs classified as “mild”, “moderate,” or “severe” in the study population before and 370 pAMIs after withdrawal. Before detoxification, 4.3% (12 out of 278) of pAMIs were classified as severe, whereas after detoxification, 3.8% (14 out of 370) were classified as severe. Additionally, before detoxification, 89.9% (250 out of 278) of pAMIs were classified as moderate, compared to 92.2% (341 out of 370) after detoxification. In addition, before detoxification, 5.8% (16/278) of pAMIs were classified as “mild”, whereas after detoxification, this proportion decreased to 4.1% (15/370) of pAMIs. In terms of severe pAMIs before detoxification, buprenorphine constituted the most frequently prescribed drug, accounting for 33.3% (4/12) of cases. Following withdrawal treatment, buprenorphine remained predominant, representing 42.9% (6/14) of cases. For moderate interactions pre-detoxification, ramipril was most commonly associated, comprising 8.8% (22/250) of cases. After withdrawal treatment, the relative proportion of ramipril decreased, but it remained the most frequently prescribed drug in this category (7.3%; 25/341). Quetiapine was identified as the primary cause of mild interactions before withdrawal treatment (93.8%; 15/16). Subsequent to withdrawal treatment, quetiapine remained prevalent, accounting for 93.3% (14/15) of cases (Table 2).

**Table 2:** Potential alcohol-medication interactions

	Before withdrawal treatment		After withdrawal treatment	
pAMIs	*n*	%	*n*	%
Total pAMIs	278	100	370	100
**Severe pAMIs**	**12**	**100**	**14**	**100**
Buprenorphine	4	33.3	6	42.9
Gabapentin	3	25.0	3	21.4
Metformin	3	25.0	2	14.3
Hydromorphone	1	8.3	2	14.3
Morphine	1	8.3	1	7.1
**Moderate pAMIs**	**250**	**100**	**341**	**100**
Ramipril	22	8.8	25	7.3
Insulin	17	6.8	20	5.9
Levetiracetam	17	6.8	17	5.0
Acetylsalicylic acid	16	6.4	16	4.7
Bisoprolol	15	6.0	17	5.0
Mirtazapine	13	5.2	24	7.0
Candesartan	9	3.6	9	2.6
Simvastatin	8	3.2	8	2.3
Atorvastatin	7	2.8	8	2.3
Pregabalin	7	2.8	7	2.1
Sertraline	7	2.8	18	5.3
Torasemide	7	2.8	10	2.9
Bupropion	6	2.4	12	3.5
Spironolactone	6	2.4	8	2.3
Escitalopram	5	2.0	1	0.3
Magnesium	5	2.0	7	2.1
Promethazine	5	2.0	7	2.1
Chlortalidone	4	1.6	4	1.2
Empagliflozin	4	1.6	3	0.9
Ibuprofen	4	1.6	5	1.5
Sitagliptin	4	1.6	5	1.5
Metoprolol	3	1.2	3	0.9
Amitriptyline	3	1.2	2	0.6
Clonidine	3	1.2	3	0.9
Dapagliflozin	3	1.2	2	0.6
Duloxetine	3	1.2	4	1.2
Hydrochlorothiazide	3	1.2	3	0.9
Methylphenidate	3	1.2	4	1.2
Valerian	3	1.2	8	2.3
Aripiprazole	2	0.8	4	1.2
Biperiden	2	0.8	1	0.3
Doxepin	2	0.8	6	1.8
Dulaglutide	2	0.8	2	0.6
Eplerenone	2	0.8	2	0.6
Fluoxetine	2	0.8	3	0.9
Valproate	2	0.8	2	0.6
Venlafaxine	2	0.8	4	1.2
Valsartan	2	0.8	3	0.9
Furosemide	1	0.4	1	0.3
Lercanidipine	1	0.4	1	0.3
Cariprazine	1	0.4	0	00
Carvedilol	1	0.4	2	0.6
Daridorexant	1	0.4	7	2.1
Diclofenac	1	0.4	1	0.3
Haloperidol	1	0.4	0	0.0
Levodopa	1	0.4	1	0.3
Lithium	1	0.4	2	0.6
Methocarbamol	1	0.4	1	0.3
Metoclopramide	1	0.4	0	0.0
Naltrexone	1	0.4	9	2.6
Nebivolol	1	0.4	1	0.3
Olanzapine	1	0.4	3	0.9
Rosuvastatin	1	0.4	1	0.3
St. John’s wort	1	0.4	0	0.0
Tamsulosin	1	0.4	3	0.9
Trimipramine	1	0.4	0	0.0
Verapamil	1	0.4	1	0.3
Enalapril	1	0.4	1	0.3
Trazodone	0	0	5	1.5
Lisdexamfetamine	0	0	3	0.9
Cetirizine	0	0	2	0.6
Desvenlafaxine	0	0	2	0.6
Oxazepam	0	0	2	0.6
Diazepam	0	0	1	0.3
Doxazosin	0	0	1	0.3
Propanolol	0	0	1	0.3
Risperidone	0	0	1	0.3
Dihydralazine	0	0	1	0.3
**Mild pAMIs**	**16**	**100**	**15**	**100**
Quetiapine	15	93.8	14	93.3
Abacavir	1	6.2	1	6.7

*Abbreviations* pAMI, potential alcohol-medication interaction

### Potential drug–drug interactions

Of the 153 patients, 19.0% experienced at least one pDDI before withdrawal treatment, rising to 22.9% after withdrawal treatment (*p* < 0.001 (McNemar test)). A total of 76 pDDIs were identified both before and after the inpatient stay, categorised as moderate, severe or contraindicated. Notably, the combination of levodopa and olanzapine, a contraindicated pairing, was present in the population both before and after withdrawal treatment.

Before detoxification, 44.7% (34/76) of pDDIs were considered severe, while after detoxification, 40.8% (31/76) were considered severe. The most common pDDI categories before detoxification were “increased risk of renal failure” (35.5%; 27/76), “pharmacokinetic interaction” (18.4%; 14/76) and “pharmacodynamic interaction” (15.8%; 12/76). After detoxification, these categories remained prevalent with “increased risk of renal failure” in 35.5% (27/76), “pharmacodynamic interaction” in 17.1% (13/76) and “pharmacokinetic interaction” in 15.8% (12/76).

The study found that before detoxification, the most common severe pDDIs were chlortalidone + ramipril and ramipril + spironolactone (both 8.8%; 3/34). After detoxification, the most common severe pDDIs were ramipril + spironolactone (16.1%; 5/31) and chlortalidone + ramipril (9.7%; 3/31). For moderate pDDIs, the combination of acetylsalicylic acid + ramipril was the most common before withdrawal treatment (9.8%; 4/41) and remained the most common after treatment (11.4%; 5/44) (Table 3).

**Table 3:** Potential drug-drug interactions

	Before withdrawal treatment		After withdrawal treatment	
pDDIs	*n*	%	*n*	%
Total pDDIs	76	100	76	100
**Severe pDDIs**	**34**	**100**	**31**	**100**
Chlortalidone - Ramipril	3	8.8	3	9.7
Ramipril - Spironolactone	3	8.8	5	16.1
Acetylsalicylic acid - Chlortalidone	2	5.9	2	6.5
Acetylsalicylic acid - Ibuprofen	2	5.9	1	3.2
Acetylsalicylic acid - Spironolactone	2	5.9	2	6.5
Amisulpride - Thioridazine	1	2.9	0	0.0
Amitriptyline - Clonidine	1	2.9	0	0.0
Amitriptyline - Sertraline	1	2.9	1	3.2
Apixaban - Acetylsalicylic acid	1	2.9	1	3.2
Acetylsalicylic acid - Candesartan	1	2.9	1	3.2
Acetylsalicylic acid - Escitalopram	1	2.9	0	0.0
Acetylsalicylic acid - Furosemide	1	2.9	0	0.0
Acetylsalicylic acid - Torasemide	1	2.9	1	3.2
Atorvastatin - Cobicistat	1	2.9	1	3.2
Bisoprolol - Clonidine	1	2.9	1	3.2
Candesartan - Torasemide	1	2.9	1	3.2
Ciprofloxacin - Sucralfate	1	2.9	0	0.0
Citalopram - Metoclopramide	1	2.9	0	0.0
Diclofenac - Escitalopram	1	2.9	0	0.0
Donepezil - Haloperidol	1	2.9	0	0.0
Doxepin - Venlafaxine	1	2.9	1	3.2
Eplerenone - Ramipril	1	2.9	1	3.2
Escitalopram - Quetiapine	1	2.9	0	0.0
Ibuprofen - Candesartan	1	2.9	0	0.0
Ibuprofen - Spironolactone	1	2.9	0	0.0
Ibuprofen - Torasemide	1	2.9	2	6.5
Ramipril – Furosemide	1	2.9	0	0.0
Doxepin - Sertraline	0	0	2	6.5
Candesartan - Ibuprofen	0	0	1	3.2
Dexamethasone - Ibuprofen	0	0	1	3.2
Diclofenac - Sertraline	0	0	1	3.2
Ibuprofen - Sertraline	0	0	1	3.2
Movicol - Spironolaction	0	0	1	3.2
**Moderate pDDIs**	**41**	**100**	**44**	**100**
Acetylsalicylic acid - Ramipril	4	9.8	5	11.4
Amlodipine - Simvastatin	3	7.3	3	6.8
Levomethadone - Pregabalin	3	7.3	3	6.8
Amlodipine - Ibuprofen	2	4.9	2	4.5
Acetylsalicylic acid - Metamizole	2	4.9	3	6.8
Dapagliflozin - Torasemide	2	4.9	2	4.5
Iron - Pantoprazole	2	4.9	2	4.5
Ibuprofen - Ramipril	2	4.9	1	2.3
Allopurinol - Chlortalidone	1	2.4	1	2.3
Amitriptyline - Levodopa	1	2.4	1	2.3
Amlodipine - Bisoprolol	1	2.4	1	2.3
Amlodipine - Metamizole	1	2.4	2	4.5
Apixaban - Metamizole	1	2.4	1	2.3
Acetylsalicylic acid - Valsartan	1	2.4	1	2.3
Atorvastatin - Colecalciferol	1	2.4	1	2.3
Dapagliflozin - Insulin	1	2.4	0	0.0
Enalapril - Furosemide	1	2.4	1	2.3
Furosemide - Metformin	1	2.4	1	2.3
Gabapentin - Hydromorphone	1	2.4	1	2.3
Haloperidol - Venlafaxine	1	2.4	0	0.0
Lercanidipine - Metoprolol	1	2.4	1	2.3
Levodopa - Sertraline	1	2.4	1	2.3
Lithium - Pipamperone	1	2.4	2	4.5
Metoprolol - Moxonidine	1	2.4	0	0.0
Mirtazapine - Sertraline	1	2.4	2	4.5
Mycophenolate Mofetil - Pantoprazole	1	2.4	1	2.3
Quetiapine - Valproate	1	2.4	0	0.0
Ramipril - Torasemide	1	2.4	1	2.3
Sertraline - Valproate	1	2.4	1	2.3
Algeldrat - Dolutegravir	0	0	1	2.3
Bupropion - Sertraline	0	0	1	2.3
Levetiracetam - Pregabalin	0	0	1	2.3
**Contraindicated combination**	**1**	**100**	**1**	**100**
Levodopa - Olanzapine	1	100	1	100

## Discussion

The present study investigated the influence of inpatient withdrawal treatment on the probability of prevalence and characteristics of pAMIs and pDDIs in general in patients treated for AUD at the addiction unit of a university hospital in Germany over a period of one year. Two different tools to detect potential drug interactions were used, namely the drugs.com classification for pAMIs and the interaction program AiD*Klinik*^®^ for the detection of pDDIs. To our knowledge, this is the first study to apply the drugs.com classification to patients with AUD and to investigate the impact of inpatient withdrawal treatment on the risk of pAMIs.

Our study population differed from previous studies regarding age, gender and comorbidity profiles [19–21]. The median age of our study population was 47 years, and the most prevalent psychiatric diagnosis besides alcohol use disorder was depression. Previous studies have investigated the prevalence and characteristics of pAMIs within the general population [14, 17]. These studies consistently indicate that a significant portion of individuals are prescribed medications that potentially interact with alcohol [14, 17]. The prevalence of such prescriptions varies widely, ranging from 13 to 42%, likly due o differences in study designs and settings [14, 17]. The most commonly prescribed drugs involved in pAMIs include benzodiazepines, antipsychotics, and medications for the treatment of cardiovascular diseases [14, 17]. 

Many studies have investigated the characteristics of pAMIs in geriatric populations [22–25]. A systematic review by Holton et al. reported that 21–35% ofolder adults may be affected by pAMIs [20]. In addition, Schröder et al. (2024) identified potentially significant interactions between alcohol and prescribed medications in over 80% of acohort of geriatric inpatients with AUD [21]. 

In the present study inpatient withdrawal treatment appeared to increase the risk of patients to be affected by at least one pAMI.

For severe pAMI, the most commonly prescribed medications in our population before withdrawal treatment were buprenorphine, gabapentin and metformin. After withdrawal treatment, the most commonly prescribed drugs were buprenorphine, gabapentin and metformin. Opioid prescriptions are common among patients with AUD, often as a medication for coexisting opioid use disorder or for analgesia [26]. In our study, 11.5%of the patients had an opioid use disorder. Jobski et al. (2015) reported frequent pharmacokinetic interactions between orally administered opioids and alcohol, leading to a significant increase in ADR incidence, highlighting the clinical relevance of this combination [27]. The use of gabapentin with alcohol appears to increase the risk of tachycardia [28]. In addition, the prescription of biguanides such as metformin to patients with AUD significantly increases the risk of potentially fatal lactic acidosis and is therefore contraindicated [29, 30]. 

For moderate pAMIs, the most commonly prescribed drugs before withdrawal treatment were ramipril, insulin and levetiracetam. After withdrawal treatment, the most commonly prescribed drugs were ramipril, mirtazapine and insulin. Alcohol consumption can acutely lower blood pressure and, when combined with antihypertensive drugs such as ramipril, increases the risk of hypotension, which can lead to serious falls [31]. Hypoglycemia is not uncommon in patients with AUD treated with insulin due to poor nutritional status or impaired liver function, so any blood glucose-lowering prescription should be carefully checked [32]. Patients with AUD are at increased risk of withdrawal seizures, but long-term prescription of antiepileptic drugs without a formal diagnosis of epilepsy should be critically evaluated due to lack of regulatory approval [33]. In particular, levetiracetam, although commonly prescribed, is not appropriate for long-term treatment of AUD because it may increase alcohol consumption [34]. The frequent prescription of mirtazapine after withdrawal treatment may be due to the many psychiatric comorbidities in this population. Mirtazapine, a sedating antidepressant, has adverse effects that are potentiated by alcohol, although the exact mechanisms remain poorly understood [35]. 

For mild interactions before withdrawal, the most commonly prescribed drugs were quetiapine and abacavir. After withdrawal, the prescriptions remained the same: quetiapine and abacavir. The sedative effect of quetiapine can lead to synergistic effects when taken with alcohol [36]. Abacavir is metabolized by alcohol dehydrogenase, leading to reduced formation and elimination of its metabolites in patients with impaired liver function, making it contraindicated in patients with moderate to severe hepatic impairment, which is common in patients with AUD [37, 38]. 

The results of our study suggest that a significant proportion of medications prescribed to patients with AUD should be critically evaluated. Although the drugs.com classification system was not developed specifically for patients with AUD, it may be a valuable tool in improving medication safety in this population. Using the drugs.com classification criteria can guide a comprehensive evaluation of prescribed medications for patients with AUD. This process requires a thorough analysis of the benefits and risks of each medication, as well as careful consideration of alternative pharmacological and non-pharmacological options.

Inpatient withdrawal treatment appeared to increase the risk for patients to be affected by at least one pDDI (*p* < 0.001). The most common pDDI category before withdrawal treatment was “increased risk of renal failure”, and this category remained prevalent after withdrawal treatment with the same proportion. In a study by Schröder et al. (2024), the most common pDDI categories in a cohort of older patients with AUD were “hypotension” and “electrolyte disturbances” [21]. 

In our study, chlortalidone + ramipril and ramipril + spironolactone were the most common serious pDDIs before withdrawal treatment. Following inpatient treatment, the most common serious pDDIs were ramipril + spironolactone and chlortalidone + ramipril. Despite the relative safety of many antihypertensives, they are still responsible for a relevant number of DDIs and ADRs [39]. When spironolactone and ACE inhibitors are combined, attention should be paid to the increased risk of hyperkalemia [40]. In patients with volume depletion, which is particularly common in alcohol-dependent individuals, the use of thiazide(-like) diuretics such as chlorthalidone can exacerbate this condition [41]. When ACE inhibitors are used concomitantly, this combination can lead to an excessive drop in blood pressure and symptomatic hypotension [42]. 

For moderate pDDIs, the combination of acetylsalicylic acid + ramipril was the most frequently observed combination before withdrawal treatment. After inpatient treatment, this combination remained the most common moderate pDDI. There is evidence that acetylsalicylic acid reduces the improvement in glomerular filtration and renal plasma flow induced by ACE inhibitors and should therefore only be prescribed under close monitoring of renal function [43]. 

Levodopa + olanzapine was the only contraindicated combination prescribed both before and after withdrawal treatment. In patients with movement disorders treated with levodopa, olanzapine has such a detrimental effect on motor symptoms that its use is contraindicated [44]. 

In conclusion, our study found that after inpatient withdrawal treatment, a significantly higher proportion of patients with AUD were treated with medications that may interact with alcohol or other drugs. The use of drug assessment tools, such as the drugs.com classification and the AiD*Klinik*^®^ interaction program, appears to be useful in clinical practice to improve drug safety in patients with AUD. A caveat of these drug assessment tools is that they were not developed specifically for patients with substance use disorders. Of note, the drugs.com classification and the AiD*Klinik*^®^ interaction program do not propose more appropriate (non-)pharmacological alternatives.

One might question the selection of the drugs studied in our study. The medications were taken from the patients’ prescriptions before and after inpatient treatment. The therapeutic goal is to achieve alcohol abstinence after qualified withdrawal therapy, so that in theory there should be no interaction between alcohol and medications after discharge from hospital. Unfortunately, relapse to alcohol use after discharge is not uncommon; therefore, alcohol-medication interactions are a relevant problem in clinical practice that healthcare providers should be able to address appropriately [45]. 

Limitations of our study are the monocentric design and the setting in a highly specialized unit of a university hospital; therefore, our results may not be fully applicable to other healthcare settings. Furthermore, due to the design of our study, we were not able to investigate whether the pAMIs or pDDIs detected in our study population actually led to the occurrence of adverse effects. Future research should focus on analyzing the true risk of adverse outcomes associated with pAMIs and pDDIs in patients with AUD. This will help healthcare professionals to stratify patients with AUD according to their individual risk profile at the time of prescribing.

## Data Availability

The data that support the findings of this study are available upon reasonable request from the corresponding author.

## Abbreviations

ADR: Adverse Drug Reaction
AMI: Alcohol-Medication Interaction
DDI: Drug-Drug Interaction
pAMI: Potential Alcohol-Medication Interaction
pDDI: Potential Drug-Drug Interaction
